# Keep the bedtime story: A daily reading ritual improves empathy and creativity in children

**DOI:** 10.1371/journal.pone.0340068

**Published:** 2026-01-09

**Authors:** Maeve Winter, A. J. Willy, James Ingersoll, Michael Joseph Meyer, Erin B. D. Clabough

**Affiliations:** 1 Department of Psychology, University of Virginia, Charlottesville, Virginia, United States of America; 2 Department of Biology, Hampden-Sydney College, Hampden-Sydney, Virginia, United States of America; 3 Program in Fundamental Neuroscience, University of Virginia, Charlottesville, Virginia, United States of America; Public Library of Science, UNITED KINGDOM OF GREAT BRITAIN AND NORTHERN IRELAND

## Abstract

Creativity and empathy are interconnected skills that have shown concerning declines in young people, yet both can be enhanced through practice-based interventions. We examined whether a two-week nightly bedtime reading routine could improve empathy and creativity in children aged 6–8 (N = 38). Participants were randomly assigned to either read picture books straight through or pause at a conflict point to ask two reflection questions about characters’ feelings and potential actions. Children completed measures of empathy (modified Interpersonal Reactivity Index) and creativity (Wallach-Kogan Alternative Uses Test) before and after the intervention. Both groups showed significant improvements in cognitive empathy, total empathy, creative fluency, and creative originality, regardless of the reflection condition. However, children in the pause group demonstrated significantly greater gains in creative fluency compared to the read-through group. Older children showed lower creative originality than younger children, with no sex differences observed. These findings suggest that consistent bedtime reading—with or without structured reflection—may enhance empathy and creativity in young children, providing families with an accessible intervention to counter societal declines in these critical skills.

## Introduction

Empathy is a complex construct that plays an important role in learning and connecting with others [1]. Multifaceted empathy develops early on and expands as we grow, nurtured by various implicit and explicit experiences and exchanges [2,3]. Self-reported empathy in American college students and medical students has dramatically declined over the last 30 years, particularly on measures of empathetic concern and perspective taking [4,5]. This decline could be due to environmental factors present in today’s society, including recent changes in technology use, child-rearing practices, education and lifestyle differences. This decline is concerning because reduced empathy makes it more difficult to connect with others and may be contributing to the growing sense of loneliness and social disconnection in our society [6]*.*

Empathy can be categorized as cognitive empathy/perspective-taking and emotional/affective empathy [1]. Cognitive empathy is related to the ability to understand the ways others think. This ability to recognize another person’s emotional or cognitive state is related to perspective taking and Theory of Mind and involves an appreciation of the other’s situation without necessarily becoming emotionally involved [7]. Emotional empathy is related to the ability to feel what others feel. It is the automatic response to another person’s emotion [8]. Empathy, which encompasses both cognitive and emotional aspects, plays a pivotal role in achieving emotional, social, and academic success. By integrating essential social and emotional dimensions into the learning process, empathy contributes to the development of a holistic individual. Even very young children demonstrate empathetic behavior. This starts with emotional contagion in infancy, and by two years old, nearly all children will engage in some sort of helping behavior if someone is distressed, giving hugs and asking, ‘Are you okay?’ [9]. Research has demonstrated that cognitive empathy, and to a smaller extent, affective empathy, are both linked to theory of mind [7]. The simultaneous development of theory of mind (ToM) and empathy during the preschool years, along with their shared neural foundations [10,11], supports this notion.

In the Western and Buddhist moral psychology tradition, empathy is considered as a projection of imagination that is linked to compassion and altruism. “Exchanging self and other” is an underemployed tool that is highly responsive to training [12]. We know that empathy is innate, but empathy levels are not solely regulated by genetics; instead, they can be altered by our everyday life experiences [13]. Research demonstrating that experience-based interventions can increase the amount of empathy a perceiver feels for a target also supports the notion that empathy is malleable—these interventions often employ perspective-taking exercises, encouraging perceivers to explicitly consider targets’ cognitive or emotional states [14].

People can spontaneously imagine other people’s thoughts or emotional processes, but we can also do this purposefully [15]. Research shows that empathy can be taught and learned with evidence-based education [16]. Specifically, spaced-out practice events that allow for repetition of concepts may be a more effective way to enhance empathy than a single longer empathy training [16]. Younger children show more gains in empathy than older children after empathy training, which is important to a discussion about ways to increase empathy in the next generation [17], and there is evidence that identification with fictional characters may be an important early step in cultivating empathy [18].

Creativity and empathy share common traits, with each relying on the other to thrive. Research indicates that, like empathy, creativity can be taught, especially through instruction that emphasizes the importance of empathizing with the subject matter [9]. One study found that in the context of engineering design empathic concern tendencies positively impacted the generation of more ideas and perspective-taking tendencies positively impacted participants’ propensity for selecting “elegant” ideas [19]. Research has found significant correlations positive between creative thinking and visualization or mental imagery with one study finding that participants’ imaging ability had significant effects on creative fluency, originality, and elaboration [20].

Research shows that the daily practice of reading to children extends beyond the scope of language development and literacy. It is a powerful tool in the cultivation of empathy from a young age. Empirical evidence suggests that consistent parental engagement in reading activities with their children significantly enhances the child’s empathy. Reading fiction might also enhance empathy, as engaging with literary fiction improves mentalizing skills and can alter how individuals perceive others’ emotions and mental states [21]. Additionally, people who read fiction are more inclined to interpret others’ mental states through the character’s perspective and to empathize with the feelings and actions of fictional characters [22].

Reflection is the process of considering situations with an element of self-awareness in order to inform future behavior [23]. Reflection plays a crucial role in fostering empathy by enabling individuals to better understand and relate to others’ emotional states [24]. Research has shown that reflective practices can enhance one’s emotional intelligence and levels of empathy [25]. This connection highlights the importance of reflective processes in developing interpersonal skills and emotional intelligence, underscoring the need for further exploration into how different types of reflection contribute to empathy.

With empathy and creativity levels declining among young people, we sought to examine whether a simple bedtime reading routine could enhance these important skills. Given that research demonstrates both empathy and creativity can be improved with practice, we designed a study to investigate whether adding reflective questions during story time would amplify the natural empathy-building effects of reading. We hypothesized that asking children to pause and consider what characters are feeling—particularly during conflict moments in stories—would provide additional practice with perspective-taking, a process that is fundamental to both understanding others and thinking creatively about problems. In this study, we examined the effects of a two-week daily reading routine on the creativity and self-reported empathy of 6–8-year-olds, comparing a standard reading practice with an intervention involving reflective questions. One group of children received a reflective intervention, which included pausing at a single conflict point in the stories to encourage discussion and pose questions, while the other group experienced the reading routine without additional prompts. Empathy levels were assessed before and after the study using a child-modified version of the Interpersonal Reactivity Index (IRI) questionnaire.

Our primary hypotheses centered on empathy outcomes: we predicted that children who paused for reflection would demonstrate greater improvements in cognitive empathy (understanding others’ thoughts) and emotional empathy (feeling what others feel) compared to children who heard the stories read straight through. We also hypothesized that the reflection group would show enhanced performance on creative tasks, since the same imaginative thinking that helps children understand a character’s situation can facilitate the generation of original ideas. We selected a two-week intervention period because it provided sufficient time for repeated exposure while remaining feasible for busy families to maintain because repeated, spaced-out practice sessions are more effective than one-time training for building empathy skills. This is also supported by current research on synaptic plasticity [26]. Ultimately, we aimed to demonstrate that something as accessible as bedtime stories—with or without reflection questions—could serve as a practical intervention for families to help counter the concerning declines in empathy and creativity observed in society.

## Methods

### Study participants

The Human Research Committee at Hampden-Sydney College approved these experiments on 2/26/2016 for all in-person sessions (24 children), and the University of Virginia Institutional Review Board for Social & Behavioral Sciences approved these experiments on 6/21/2021 under Protocol #4486 for all online data collection sessions. Recruitment and data collection only began after IRB approval was granted at each institution. Study visits for the first 24 participants occurred in college academic buildings and public library conference rooms, whereas the remaining participants had their initial and follow up meetings via Zoom due to COVID-19 precautions. Written consent obtained was from legal guardians of participants; verbal assent obtained from child participants.

The study contained 41 participants between the ages of 6 and 8 living in central Virginia and their parent or guardian (N = 82 participants). Subjects were recruited over a period of several years in two different locations in central Virginia separated by about 75 miles. Participant children came from many different types of schools and classrooms, and were recruited through Facebook posts, direct emails to area elementary schools, flyers posted on community bulletin boards, and in person recruitment outside local public libraries, museums, and child weekend sporting events in the areas. The diverse recruitment strategies and geographic distribution of participants across multiple educational settings was designed to capture the natural variation present in real-world family reading practices. The mean age of the child participants was 6.8 + /- 0.12 years old; there were no significant age differences between groups. (See Table 1 for demographics.) We did not collect data about the type of schooling the child had (private, public, or homeschooled).

**Table 1:** Demographic information of study participants.

Variables Description	Count	Proportion	Count, Read Through	Count, Pause
**Grade**				
Kindergarten	2	5.26%	0	2
1^st^ Grade	19	50.0%	8	11
2^nd^ Grade	13	34.21%	7	6
3^rd^ Grade	4	10.52%	4	0
**Gender**				
Male	19	50.0%	10	9
Female	19	50.0%	9	10
**Annual Family Income**				
Less than $25,000	0	0.00%	0	0
$25,000-$50,000	3	7.89%	2	1
Greater than $50,000	35	92.11%	17	18
**Reading Ability Status**				
Yes, the child can read well independently	18	47.37%	10	8
The child is a beginning reader and is somewhat independent	20	52.63%	9	11
The child cannot read independently yet	0	0.00%	0	0

### Empathy and creativity measures

We assessed self-reported empathy using a version of the Interpersonal Reactivity Index (IRI) previously used with adults [1] and children [27], further refined by Garton and Gringart for use in 8- and 9-year-olds and validated in 413 schoolchildren [28]. We used the two-factor Garton and Gringart 12-item scale [Table 3 in [28]], where Factor 1 has six cognitive empathy questions and Factor 2 has six emotional empathy questions, with two modifications. First, we included two additional questions from the IRI Fantasy sub-scale specifically about story characters. The original IRI designed for adults had two cognitive subscales: 1) perspective taking and 2) fantasy, and two emotional subscales: 3) empathic concern and 4) personal distress [1]. When Garton and Gringart carefully validated these measures in 8–9 year olds, analysis allowed the personal distress items to be redistributed and equally loaded onto those 2 factors, cleanly separating items into a two factor measure of cognitive and emotional empathy, but discarding the Fantasy items (which describe the likelihood that a person identifies with a fictional character) [28]. Although Davis’ experimental work took a similar direction after the development of the IRI as well [29], these story fantasy questions are highly applicable to our study design. Therefore, we included two questions from Litvak and colleagues’ children’s empathy measure [27] that specifically assessed perspective-taking with fictional characters, as this directly related to the skills our study children were practicing during story reading. These questions were: “When reading a book, I try to imagine what the people in the story are thinking” and “It is easy for me to pretend that I am the star of my favorite movie”.

Second, we simplified the paper and pen answer scale from ‘Not like me at all’ through to ‘Hardly ever like me,’ ‘Occasionally like me,’ ‘Fairly like me,’ and ‘Very like me’ to a verbal response of ‘Yes,’ ‘No,’ or ‘Sometimes’ for the 6–8-year-olds in our study. Garton and Gringart’s measurement scale (designed for 8–9 year olds) was not linear because some (but not all) points on the 5-point scale included a temporal element (‘hardly ever’ and ‘occasionally’) [28]. The simplification of our scale to a ‘Yes,’ ‘No,’ or ‘Sometimes’ attempts to eliminate this potential issue for younger children (aged 6–8 years) that cannot differentiate easily between the terms ‘occasionally like me’ and ‘fairly like me.’ In our study, ‘Yes’ was scored as 2, ‘Sometimes’ was scored as 1, and ‘No’ was scored as 0.

Our decision to simplify the empathy scale response format from the original five-point Likert scale to a three-point “Yes/No/Sometimes” format was driven by developmental considerations and empirical evidence from previous research with young children. Garton and Gringart (2005) identified significant methodological concerns when adapting the adult IRI for 8–9 year olds, noting that their five-point scale was “not linear because some (but not all) points on the 5-point scale included a temporal element (‘hardly ever’ and ‘occasionally’)” and concluded that children had difficulty differentiating between terms like “occasionally like me” and “fairly like me” (p. 24). Extensive research demonstrates that children ages 6–8 perform significantly better with simplified response formats due to developmental limitations in concrete operational thinking, executive function constraints, and limited metacognitive awareness required for complex self-report measures [30,31] Coombes et al. (2021) conducted a systematic review of 81 studies with over 13,000 participants and established that children ≤7 years “think dichotomously so need two response options” while children >8 years “can reliably use a 3-point scale” [32]. Our simplified three-point scale (scored as No = 0, Sometimes = 1, Yes = 2) eliminates measurement error from inappropriate scale complexity while maintaining the ability to capture meaningful variation in empathic responses developmentally appropriate for this age group.

The empathy measures were administered verbally in individual, one-on-one sessions with trained research assistants. This administration format was chosen because it allowed research assistants to ensure that each child understood the questions and could provide appropriate responses. Research assistants were trained to present each question clearly and to rephrase questions using age-appropriate language if a child appeared confused, while maintaining the core meaning of each item, which very rarely happened. The three-point response format (“Yes,” “No,” “Sometimes”) was presented verbally and each session was conducted in a quiet, comfortable setting where children could focus on the questions without distraction.

Creativity was assessed using the Wallach and Kogan alternative uses verbal test in children, specifically examining responses for fluency and originality in divergent thinking [33].

### Initial visit experience

Participants attended individual initial appointments, where the participant was randomized to a treatment group before each initial visit based on a random number generator to prevent selection bias. The child attended the initial session with a caregiver, where informed consent was obtained from both the child and caregiver. Participants were told they were participating in a study examining the impact of reading to children. At the initial visit, a study team member presented a short PowerPoint presentation that highlighted the benefits of reading aloud to your child, backed by research showing it improves school readiness and vocabulary [34,35]*.* The terms empathy and creativity were not mentioned during the presentation.

The parents of the participants were randomly assigned to the “read through” or the “pause” group and given packs of books accordingly. Engaging picture books were chosen that would likely be new to the children, but they were typical storybooks (not focused on an overt theme of empathy). The books we used were as follows: *A Letter for Leo* by Sergio Ruzzier *(*isbn: 9780544223608), *A New Friend for Marmalade* by A.M. Reynolds (isbn: 9781481420464), *Cub’s Big World* by Sarah Thomson (isbn: 9780544057395), *Library Lion* by Michelle Knudsen (isbn: 9780763637842), *Nugget & Fang* by Tammi Sauer (isbn: 9780547852850), *Stuck with the Blooz* by Caron Levis (isbn: 9780547745602), *The Tooth Fairy Wars* by Kate Coombs (isbn: 9781416979159). Selected short stories had some form of social conflict, such as a small polar bear getting separated from his mother, the tooth fairy fighting with a boy who wants to keep his lost tooth, or shark and minnow best friends who realize society says they should not be friends.

The read through group was given unaltered books, while the pause group was given books containing a small sticker with two question prompts placed in each book at a single story conflict point. Questions read “What do you think _(insert main character’s name)__ is feeling right now?” and “What would you do if you were _(insert main character’s name)__?”. Names were changed in each book to match the character in the specific story conflict. Parents in the pause group were instructed to ask the questions one at a time to their child. If the child responded, the parent just listened before continuing to read the story. If the child did not respond, the parent waited 30 seconds before reading further. Parents were instructed not to ask follow-up questions or to offer commentary on child responses.

The initial visit presentation included short video models of a mother and child sitting on a bed reading the picture book *Are You My Mother* by P.D. Eastman aloud (isbn: 9780679890478). The video for the pause group demonstrated how to read the questions “What do you think Baby Bird is feeling right now?” and “What would you do if you were Baby Bird?” and wait for answers, whereas the read through video showed a mother reading the story to a child as normal (S1 Video). Following the presentation, a study team member administered the Garton and Gringart modified IRI test and the Wallach and Kogan alternative uses test (Q1 uses for a paperclip, Q2 things with wheels) to the child [28,33], while the caregiver completed a survey with demographic information.

### Study instructions and follow up

Both groups were instructed to read each book just twice over the course of the two-week study period and to put the books away while it was not study time to prevent unequal exposure. Book readings were logged by participants in a Reading Log to ensure groups followed study instructions. After two weeks elapsed, the participants were brought back for a follow up meeting where the same tests were administered (identical modified IRI and Wallach/Kogan based on Q1 uses for a newspaper, Q2 things that are round). All participants received the seven books used for the study for free. At the conclusion of the study, three participants were drawn at random to receive $50 Barnes and Noble gift cards.

We selected a two-week intervention period to balance several competing considerations. This timeframe provided families with sufficient time to complete 14 reading sessions (seven books read twice each) while maintaining a manageable commitment that would support study completion. Pilot testing with families indicated that a two-week daily reading commitment felt achievable while still requiring meaningful engagement. Longer intervention periods would have increased participant burden and likely reduced completion rates. Since research on brief empathy interventions suggests that focused, short-term interventions can produce measurable changes in empathy-related outcomes, particularly in children who are still developing these skills, we designed our intervention to test whether even a brief modification to typical reading practices could show promise as a practical, family-friendly approach to enhancing empathy and creativity.

### Data analysis

#### Empathy measures.

Our version of the Gringart and Garton IRI Test was administered across two visits. The Gringart and Garton IRI version has no reverse coded items because they were confusing to young participants [29]. Empathy questions were scored 0 (no), 1 (sometimes), or 2 (often). Higher scores represent a presence of greater empathy in the participant. Total empathy is defined as the combined scores of emotional empathy subscale, cognitive empathy subscale, and fantasy (2 additional questions) at the same visit.

We treated our three-point empathy scale (scored as No = 0, Sometimes = 1, Yes = 2) as a continuous variable for statistical analysis, following established methodological precedent in the literature. Norman (2010) provides compelling empirical evidence that parametric tests can be appropriately used with ordinal data such as Likert scales, demonstrating that these tests are generally more robust than nonparametric alternatives and yield largely unbiased results even when statistical assumptions are violated [36]. Research consistently supports treating ordinal variables with three or more categories as approximately continuous without compromising analytical validity [37,38]. Three-category scales provide sufficient variance for meaningful parametric analysis while remaining developmentally appropriate for young children’s cognitive capabilities, and this approach is standard practice in pediatric empathy research, where similar response formats are routinely analyzed as continuous variables using regression and ANOVA [39]. The equal-interval assumption underlying our 0-1-2 scoring represents a reasonable interpretation of the semantic progression from “No” through “Sometimes” to “Yes,” reflecting a natural frequency continuum that children ages 6–8 can reliably differentiate.

Three participants had data omitted from the analysis for a total of 19 subjects in each group. One pause group participant did not return for a follow up visit and therefore was removed from analysis. Two other children in the pause group (one male and one female, both aged 6 in kindergarten) answered affirmatively to all questions on the initial empathy measure on both initial and follow up tests, so their data was not retained in analysis. One participant in the read-through group responded to all measures except fantasy, and so that participant was kept for the measures they responded to. The final sample sizes for fantasy (and by extension total empathy scores) were 18 and 19 for the read-through and pause groups, respectively.

#### Creativity measures.

The Wallach and Kogan Alternative Uses Test was administered across two visits. Creative fluency was measured by the sum of the total number of responses provided for Q1 and Q2 at the same visit. The difference in total scores between the initial and follow-up visits reflected the change in creative fluency. Creative originality assessment coded and scored each response based on its frequency relative to the total number of responses. The scores were then summed for a total originality score, where lower scores indicated more original responses.

### Statistical analysis

For all measures except fantasy ratings, a series of 2 x 2 mixed ANOVA models were performed using the rstatix and stats packages in R [40] to analyze the effects of the treatment groups (Condition: pause intervention, “control” reading intervention), the two occasions (initial test, follow-up test), and their interaction had on each of the six measures. Because the range of possible fantasy ratings was small (0–4), we instead performed a cumulative link mixed effect model using the ordinal package [41] to analyze the same effects of treatment, occasion, and their interaction on the fantasy measure. Due to the high number of analyses, a Bonferroni correction was performed on all p-values in the six primary models (five mixed ANOVA models plus the cumulative link mixed effects model for fantasy), assuming a familywise error rate of four comparisons for models that focused on the four empathy measures and two comparisons for the two creativity measures.

To check assumptions for the ANOVA models, we visualized the distributions of the residuals of these models (S1 Fig) and checked for outliers using the rstatix package in R [40]. No extreme (> 3*IQR) outliers were found, and the normality and homogeneity of variance assumptions appeared sufficiently met for all ANOVA models except for those using emotional empathy and originality as measures. Due to the potential concerns with these two models, we double-checked the assumptions of the models using statistical assumption testing methods. More specifically, we performed Shapiro-Wilk on the residuals of the models to check normality, and performed Levene’s tests at each occasion to check homoscedasticity across the intervention groups, using the stats and car packages in R, respectively [42]. When doing so, no models had significant Levene’s test results (meaning that our assumption of homoscedasticity was met), but the model that used emotional empathy had a significant Shapiro-Wilk result (Shapiro-Wilk, p = .027). However, when performing a Box-Cox analysis (using the car package in R) to determine possible transformations, the suggested lambda of 1.41 suggested no transformation should be performed. Thus, taken holistically with the visual assumption checks, no power transformations on the data were deemed necessary to use for any measures. Because our within-subjects measure (Occasion) only had two levels, sphericity was automatically met, and no assumption test on sphericity was necessary.

Following data collection, we conducted a sensitivity analysis to determine the minimum effect sizes our study was powered to detect. This post-hoc power analysis informed interpretation of both significant and non-significant findings by clarifying which effect sizes our design could reliably detect versus those for which we lacked adequate statistical power.

## Results

The majority of families reported that they regularly read to their children before the study period (64.8% read daily, 19.4% read 3-6x/week, 11.1% read 1-3x/week, and 5.6% rarely read), though it is unknown if this practice was in the same format as the study.

**Fig 1:**
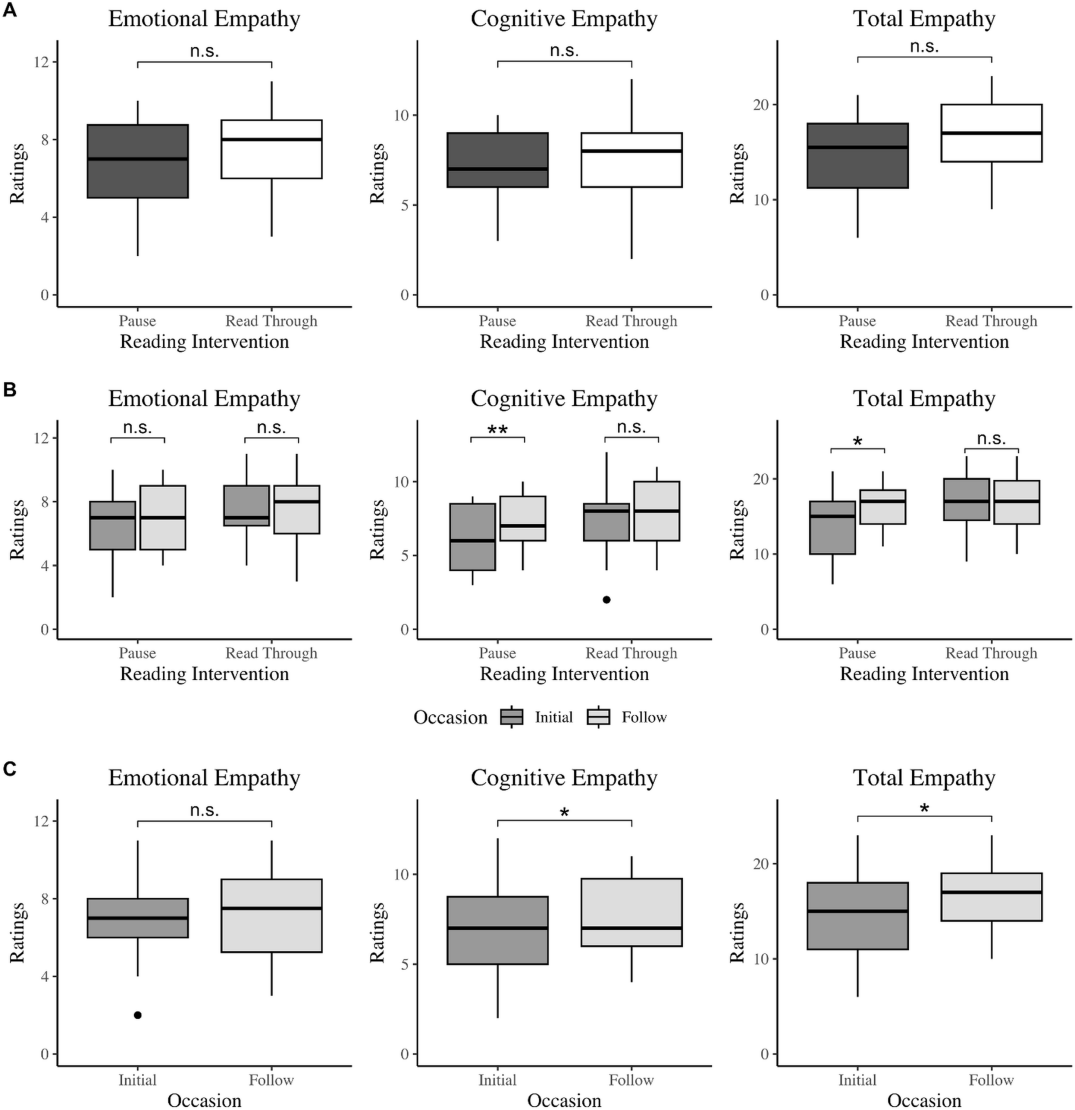
The effect of two weeks of daily picture book reading on empathy in 6-8 year-olds. **a)** Boxplots depicting differences of emotional, cognitive, and total empathy scores across the two reading intervention groups. Total empathy scores were computed as the sum of the emotional, cognitive, and fantasy empathy scores. There were no statistically significant differences in any of the three empathy scores between the intervention (pause) reading group and the control (read through) reading group (Two-way Mixed ANOVA, emotional: p = .528, cognitive: p > .999, total: p = .520). Higher scores represent a presence of greater empathy. b) and **c)** Boxplots depicting differences of emotional, cognitive, and total empathy scores across the two occasions. Cognitive and total empathy scores statistically significantly increased from the initial visit to the follow-up visit, regardless of which reading intervention group the participants were assigned (Two-way Mixed ANOVA, p = .020* and p.024*, respectively). There were no statistically significant differences in emotional empathy scores between the two occasions, however (Two-way Mixed ANOVA, p = .888).

**Fig 2:**
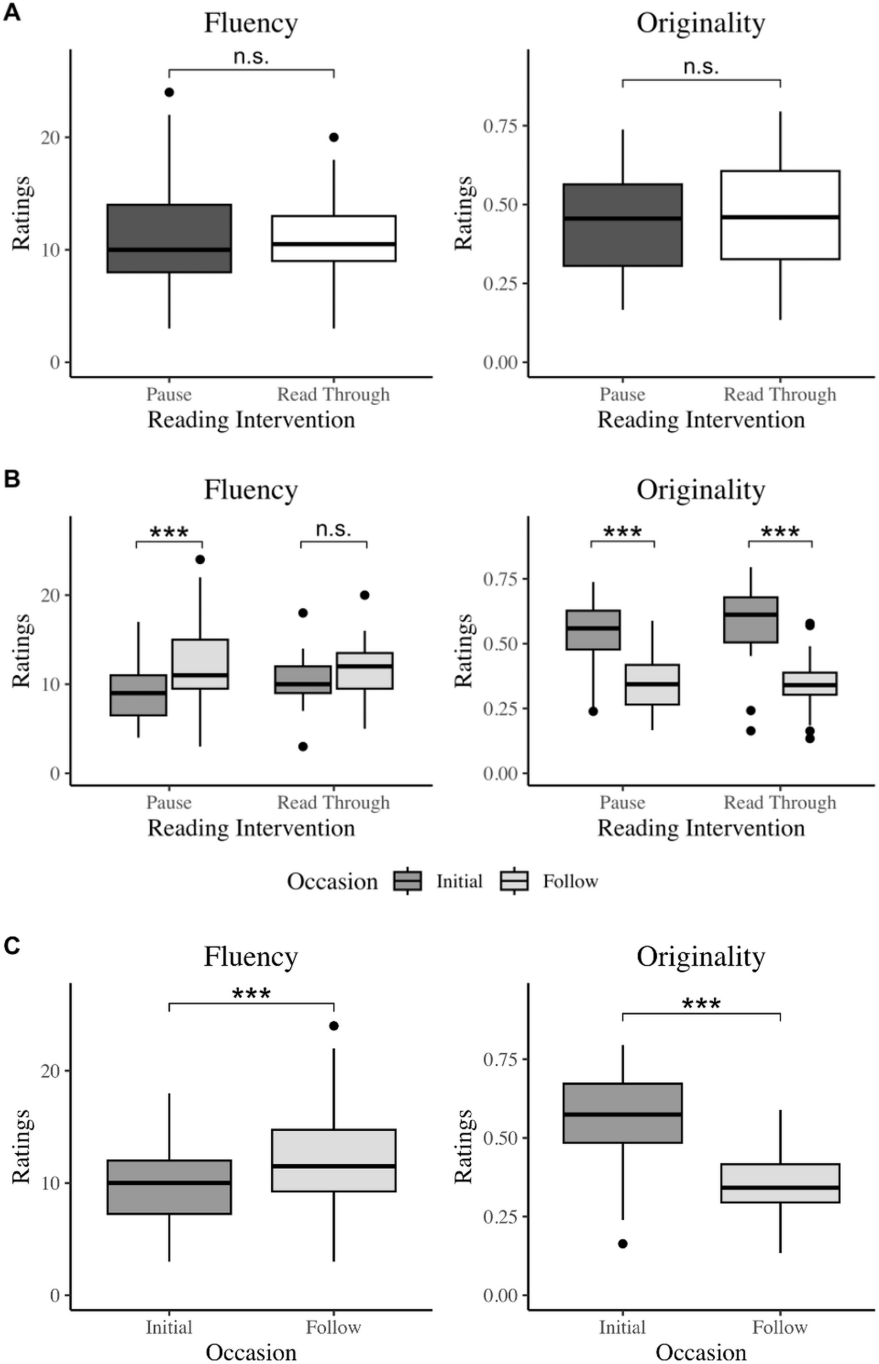
The effect of two weeks of daily picture book reading and reflection questions on creativity (fluency and originality) in 6-8 year-olds. **a)** Boxplots depicting differences of creativity (fluency and originality) scores across the two reading intervention groups. There were no statistically significant differences in either creativity score between the intervention (pause) reading group and the control (read through) group (Two-way Mixed ANOVA, fluency: p > .999, originality: p > .999). b) and **c)** Boxplots depicting differences of creativity (fluency and originality) scores across the two occasions. Both creativity types statistically significantly improved from the initial visit to the follow-up visit, regardless of reading intervention group (Two-way Mixed ANOVA, fluency: p < .001***, originality: p < .001***).

We hypothesized the participants receiving the pause reading intervention would increase performance on both empathy and creativity measures when compared to participants who did not receive the interventions. Unexpectedly, the pause intervention did not preferentially increase empathy or creativity scores between the study visits (Two-way Mixed ANOVA, p ≥ .440 across all measures; Cumulative Link Mixed Effects, p = .341 for fantasy; Figs 1 and 2; residual distributions met normality assumptions, see S1 Fig). However, significant improvement from the initial visit to the follow-up were found in almost all measures, including cognitive empathy, fantasy questions, total empathy, creativity fluency, and creativity originality (Two-way Mixed ANOVA, p ≤ .040), with the exception of emotional empathy (Two-way Mixed ANOVA, p = .888, Rp2 = 0.04) and fantasy (Cumulative Link Mixed Effects, p = .304, McFadden’s $R_{p}^{\text{2}}=\text{0}.\text{0}\text{2}$ and Nagelkerke’s $R_{p}^{\text{2}}=\text{0}.\text{0}\text{5}$), regardless of the group assigned (Figs 1 and 2).

Additionally, occasion significantly moderated the treatment effect for creativity fluency ratings (Two-way Mixed ANOVA, p = .036, $R_{p}^{\text{2}}=\text{0}.\text{14}$), meaning that children that paused for reflections showed significantly higher increases in creativity fluency when compared to the read through group (Rating difference = 3.74 ± 0.75(SE), t(36) = 4.96, p < .001, two-tailed) than for the read through group (Rating difference = 1.11 ± 0.75(SE), t(36) = 1.47, p = .151, two-tailed). This latter result suggests that, when just reading a book once, reading without pausing may lead to a small, temporary boost in higher fluency that evens out over time; in contrast, pausing while reading leads to a potentially delayed, but larger improvement in fluency after repeated readings. (See Table 2 for group means and standard deviations, Table 3 for the primary ANOVA results, and Table 4 for the primary cumulative link mixed effects model results).

**Table 2:** Descriptive statistics for Pause (Intervention) and the Read-Through (Non-Intervention) groups. Group means and standard deviations for empathy and creativity measures at initial and follow-up visits for both the pause and read-through groups, with combined group statistics provided for comparison.

	Pause (Intervention) Group		Read-Through (Non-Intervention) Group		Combined	
	Initial Visit	Follow-Up Visit	Initial Visit	Follow-Up Visit	Initial Visit	Follow-Up Visit
**Empathy measures**						
Emotional	M = 6.42	M = 6.89	M = 7.47	M = 7.74	M = 6.95	M = 7.32
	SD = 2.29	SD = 2.07	SD = 1.84	SD = 2.18	SD = 2.12	SD = 2.14
Cognitive	M = 6.26	M = 7.53	M = 7.37	M = 7.74	M = 6.82	M = 7.63
	SD = 2.13	SD = 1.74	SD = 2.34	SD = 2.35	SD = 2.28	SD = 2.05
Fantasy	M = 0.68	M = 1.95	M = 1.89	M = 1.61	M = 1.29	M = 1.78
	SD = 0.95	SD = 1.08	SD = 1.37	SD = 1.24	SD = 1.31	SD = 1.16
Total	M = 13.37	M = 16.37	M = 16.74	M = 16.89	M = 15.05	M = 16.62
	SD = 4.51	SD = 3.22	SD = 4.13	SD = 4.04	SD = 4.60	SD = 3.60
**Creativity Measures**						
Fluency	M = 9.00	M = 12.74	M = 10.37	M = 11.47	M = 9.68	M = 12.11
	SD = 3.76	SD = 5.09	SD = 3.18	SD = 3.78	SD = 3.50	SD = 4.46
Originality*	M = 0.53	M = 0.35	M = 0.58	M = 0.35	M = 0.56	M = 0.35
	SD = 0.15	SD = 0.12	SD = 0.16	SD = 0.12	SD = 0.16	SD = 0.12

*Note: Lower originality ratings equate to a higher sense of originality; for all other measures, a higher rating corresponds to a greater amount of that measure.

**Table 3:** Primary mixed ANOVA results. Results show significant main effects of occasion (initial vs. follow-up) for cognitive empathy, total empathy, creativity fluency, and creativity originality, with a significant treatment by occasion interaction for creativity fluency, indicating that children in the pause group showed greater improvements in creative fluency compared to the read-through group.

		F-Value	p-value	Corrected p-value	${\;\;R}_{p}^{\text{2}}$
**Empathy measures**					
Emotional	Treatment (Read Through vs. Pausing)	2.37	.132	.528	0.06
	Occasion (Initial vs. Follow-Up)	1.54	.222	.888	0.04
	Treatment x Occasion Interaction	0.13	.725	>.999	<0.01
Cognitive	Treatment (Read Through vs. Pausing)	1.04	.314	>.999	0.03
	Occasion (Initial vs. Follow-Up)	8.92	.005**	.020*	0.20
	Treatment x Occasion Interaction	2.68	.110	.440	0.07
Total	Treatment (Read Through vs. Pausing)	2.40	.130	.520	0.06
	Occasion (Initial vs. Follow-Up)	8.44	.006**	.024*	0.19
	Treatment x Occasion Interaction	1.92	.032*	.128	0.12
**Creativity Measures**					
Fluency	Treatment (Read Through vs. Pausing)	0.002 (<0.01)	.965	>.999	<0.01
	Occasion (Initial vs. Follow-Up)	20.69	<.001***	<.001***	0.36
	Treatment x Occasion Interaction	6.11	.018*	.036*	0.14
Originality	Treatment (Read Through vs. Pausing)	0.30	.587	>.999	0.01
	Occasion (Initial vs. Follow-Up)	80.74	<.001***	<.001***	0.69
	Treatment x Occasion Interaction	0.78	.382	.764	0.02

Note: The F-values for Total Empathy have 1 and 35 degrees of freedom, and all other F-values have 1 and 36 degrees of freedom. Rows that are statistically significant, post-correction, are shaded green. Corrected p-values are based on a Bonferroni adjustment, assuming a familywise error rate of four comparisons for models that focused on the four empathy measures (see Table 4 for the fantasy empathy measure results), and two comparisons for the two creativity measures. The number of stars after each p-value represent the level of statistical significance; * indicates 0.01≤p<0.05; ** indicates 0.001≤p<0.01; *** indicates p<0.001.

**Table 4:** Primary cumulative link mixed effects model results for fantasy measures. Results show a significant treatment by occasion interaction for fantasy empathy ratings, indicating that children in the pause group demonstrated significantly greater improvements from initial to follow-up visits compared to the read-through group.

Measures	Effect	Slope	Z-Value	p-value	Corrected p-value	$R_{p}^{2}$ (McFadden)	$R_{p}^{2}$ (Nagelkerke)
**Empathy**							
Fantasy	Treatment (Read Through vs. Pausing)	0.40	1.72	.085	.341	0.01	0.04
	Occasion (Initial vs. Follow-Up)	−0.42	−1.77	.076	.304	0.02	0.05
	Treatment x Occasion Interaction	0.64	2.47	.014*	.054	0.04	0.11

Note: Rows that are statistically significant, post-correction, are shaded green. Corrected p-values are based on a Bonferroni adjustment, assuming a familywise error rate of four comparisons for models that focused on the four empathy measures (see Table 3 for the other three empathy measure results). The reported $R_{p}^{\text{2}}$ values are McFadden and Nagelkerke pseudo R-square measures, respectively, computed using Type II model comparisons. The number of stars after each p-value represent the level of statistical significance; * indicates 0.01≤p<0.05; ** indicates 0.001≤p<0.01; *** indicates p<0.001.

While there were some differences between the two treatment groups found from the initial visit results, there were no statistically significant differences between the two treatment groups at baseline for any measures except for the fantasy ratings, after correcting for multiple comparisons using a Bonferroni adjustment. In this case, the pause group was found to have statistically significantly lower ratings than the read through group at baseline (Z-test, p=.028, [0.57, 3.56]). However, the pause group scores improved during the follow-up to the point where these differences were no longer significant (Z-test. p > .999, [−1.67, 0.71]).

Since these results may potentially differ by child demographics, we examined sex, age, and prior reading status, and age as potential moderator variables by testing three-way interactions to determine whether these factors influenced the relationship between treatment condition and empathy/creativity outcomes. We performed a series of three-way Mixed ANOVAs that included sex as a moderator, another series of three-way Mixed ANOVAs that included prior reading status as a moderator, and a series of three-way Mixed ANCOVAs that separately included age as a moderator for five of the six measures, and equivalent cumulative link ordinal models for the fantasy ratings.

When including sex as a moderator, the primary results regarding treatment and occasion effects, along with the results found at baseline, mostly held true. After controlling for sex differences, occasion significantly moderated the treatment effect when looking at fantasy ratings (Cumulative Link Mixed Effects, p=.034, McFadden’s $R_{p}^{\text{2}}=\text{0}.\text{0}\text{4}$ and Nagelkerke’s $R_{p}^{\text{2}}=\text{0}.\text{1}\text{2}$). There was a statistically significant improvement in fantasy ratings from the initial reading to the follow up for the pause group (Rating difference=2.26±0.82(SE), z = 2.75, p=.006, two-tailed), while the difference in ratings was non-significant for the read through group (Rating difference=−0.59±0.66(SE), z = 0.90, p=.366, two-tailed). Otherwise, there were no significant sex differences across both occasions and both treatment groups (Three-way Mixed ANOVA and Cumulative Link Mixed Effects, p ≥ .124) (S1 and S4 Tables).

When including prior reading as a moderator, the results again remain mostly consistent with the primary results. After controlling for prior reading differences, occasion significantly moderated the treatment effect when looking at fantasy ratings (Cumulative Link Mixed Effects, p=.038, McFadden’s $R_{p}^{\text{2}}=\text{0}.\text{0}\text{4}$ and Nagelkerke’s $R_{p}^{\text{2}}=\text{0}.\text{1}\text{2}$). There was a statistically significant improvement in fantasy ratings from the initial reading to the follow up for the pause group (Rating difference=2.32±0.83(SE), z = 2.81, p=.005, two-tailed), while the difference in ratings was non-significant for the read through group (Rating difference=−0.54±0.66(SE), z = 0.82, p=.412, two-tailed). With regards to specific prior reading differences, more independent children did significantly worse (higher ratings) on originality compared to beginner reading children across both occasions and both reading types (Three-way Mixed ANOVA, p=.014, $R_{p}^{\text{2}}=\text{0}.\text{0}\text{2}$). Outside of these notable results, there were no additional significant prior reading differences across occasion or reading type for any of the measures (Three-way Mixed ANOVA and Cumulative Link Mixed Effects, p≥.192) (S2 and S4 Tables).

**Fig 3:**
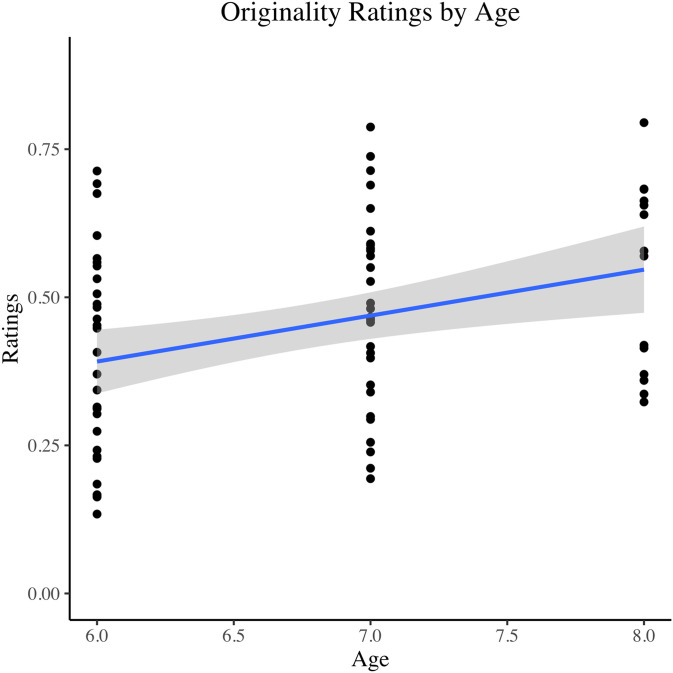
The effect of two weeks of daily picture book reading and reflection questions on originality as a measure of creativity in 6-8 year-olds. Scatterplot depicting the overall relationship between age groups and creative originality ratings. When including age as an additional moderator, older children did statistically significantly worse on creative originality compared to younger children across both occasions and both reading types (Three-way Mixed ANCOVA, p = .018) NOTE: Lower scores are more original.

When including age as a moderator, the improvement in cognitive empathy and total empathy ratings from the initial visit to the follow-up was no longer statistically significant (Cognitive: Three-way Mixed ANCOVA, p* *= .096, $R_{p}^{\text{2}}=\text{0}.\text{15}$; Total: Three-way Mixed ANCOVA, p=.184, $R_{p}^{\text{2}}=\text{0}.\text{1}\text{2}$). Likewise, the interaction effect between treatment and occasion on fluency scores was no longer statistically significant (Three-way Mixed ANCOVA, p* *= .090, $R_{p}^{\text{2}}=\text{0}.\text{12}$). With regards to specific age differences, older children did significantly worse (higher ratings) on originality compared to younger children across both occasions and both reading types (Three-way Mixed ANCOVA, p = .018, $R_{p}^{\text{2}}=\text{0}.\text{25}$) (Fig 3). Outside of these notable results, there were no additional significant age differences across occasion or reading type for any of the measures (Three-way Mixed ANCOVA and Cumulative Link Mixed Effects, p* *≥ .668), and baseline differences across treatment levels stayed consistent after controlling for age (S3 and S4 Tables).

To aid interpretation of our findings, we conducted a post-hoc sensitivity analysis. With N = 38 (N = 19 per group) and α = 0.05, our 2 × 2 mixed ANOVA design achieved 80% power to detect treatment × occasion interaction effects of partial η^2^ ≥ 0.18 (a large effect by Cohen’s guidelines). Our study had substantially lower power for smaller effects (68% for η^2^ = 0.14, 60% for η^2^ = 0.12, 47% for η^2^ = 0.09, and 33% for η^2^ = 0.06).

Our significant finding for creativity fluency (η^2^ = 0.14, p = 0.036) falls at the threshold of large effects and had 68% power. The fact that we detected this effect despite being slightly underpowered strengthens confidence in this finding. However, several empathy measures showed non-significant interactions with medium effect sizes (total empathy: η^2^ = 0.12, p = .128; cognitive empathy: η^2^ = 0.07, p = 0.440), suggesting our study was underpowered to detect these smaller effects. Thus, the absence of significant treatment × occasion interactions for most empathy measures could reflect either genuinely similar effects of both reading conditions, or smaller differential effects that our sample size could not reliably detect. The very small effect for emotional empathy (η^2^ < 0.01) likely represents a true absence of differential effects rather than insufficient power.

## Discussion

Our study provides valuable insights into how a brief but consistent daily reading practice can positively influence the development of empathy and creativity in young children.

### Fostering empathy

Our results demonstrate that both reading groups showed significant improvements in cognitive empathy and total empathy scores from initial to follow-up visits, suggesting that daily reading routines may enhance children’s empathic abilities. This finding is consistent with research suggesting that reading facilitates the development of empathy by allowing children to engage with diverse characters and perspectives. Books serve as a shared reference for discussing characters’ emotions and thoughts, providing unique opportunities for conversations about mental states [43,44]. In addition, scaffolding, such as guided discussions around storybooks, can enhance children’s ability to extract moral lessons and apply them to their own experiences [45]. Our data suggest that the scaffolding within the book storyline may be sufficient to produce empathy changes when simply read by the caregiver, even without the additional reflection prompts during the story.

We found that cognitive empathy showed significant improvement after daily picture book reading over a two-week period, while emotional empathy did not reach statistical significance. This is consistent with previous literature suggesting that cognitive empathy may be more responsive to short-term perspective-taking practice [17]. Regular reading helps children better understand and identify with others’ emotions through perspective-taking [46], and narratives featuring protagonists can aid children in adopting new perspectives. Using picture books to address social issues integrates critical literacy skills and fosters social awareness, promoting empathy [47], and may potentially boost ethnocultural empathy [2].

Emotional empathy has been traditionally harder to change over small periods of time—it may require more time to alter deep rooted ways of identifying, processing, and acting on emotions, as it involves different brain regions than cognitive empathy [48]. Future research should investigate if emotional empathy measures may be changed with longer intervention periods or if the observed cognitive empathy improvements persist over time as children move through development. The finding that pausing to reflect during stories did not produce statistically significant empathy gains compared to reading stories without pausing suggests that children may naturally engage in perspective-taking while listening to stories or that the immersive experience of continuous reading has its own benefits [21].

### Fostering creativity

Our study also found significant improvements in both creative fluency and creative originality measures after the 2-week reading experience in both groups. Creativity is often perceived as an innate talent or quality, but our results support research showing it can also be enhanced through practice, especially employing idea generation and cognitive training approaches [49]. The improvements observed in both groups could reflect genuine practice effects from the reading intervention, increased comfort with the testing procedures, or the benefits of creative practice in a supportive environment during the initial assessment.

Notably, we found that children in the pause group showed significantly greater improvements in creative fluency compared to the read-through group. This interactive scaffolding of the creative process—where children generated ideas about possible actions for story characters—represents a perspective-taking exercise that may simultaneously engage both creativity and empathetic processing. This task asked children to imagine possible storylines for the second half of the book, tapping into both creative and empathetic abilities.

Previous research examining the Alternative Uses Task in 4–6 year olds found that scores increase over this developmental period, likely because the test procedures heavily rely on children’s verbal skills, which are also developing over time [50]. However, our data revealed that older children in our sample (ages 6–8) showed lower creative originality scores than younger children, a pattern consistent with research documenting creativity declines in school-age children. Fostering creativity can be challenging in a world with rigid values, as strict rules may lead children to prioritize social acceptance over creative expression as they grow older [51]. Seminal work on creativity during development postulates a slump in creativity evident in around fourth grade [52], though this pattern varies among individual children. In fact, some children exhibit an increase in creativity during this time, with longitudinal changes in frontal neural connectivity linked to developmental improvements in creative thinking ability using functional near-infrared spectroscopy (fNIRS) [53].

The complex literature on creativity development may be the result of a modern social and educational structure that contains mixed messaging — while our culture theoretically emphasizes the importance of creative expression, assessment systems in both schools and the workforce often devalue it. The reflective reading approach in our study provides practice with imaginative and empathetic engagement through narratives, which may counteract observed age-related decreases in creative expression. Future research should examine whether these effects persist over time, as previous research showed that creativity training can still boost divergent thinking a year later [54].

### Developmental timing of interventions

We chose to investigate the development of empathy skills during a developmental period (6–8 years old) when children are first exploring reading independently, but also still undergoing intense synaptogenesis that begins before birth and extends into adolescence [55], experiencing dynamic brain changes in response to environmental exposure. This developmental stage may represent a sensitive period for interventions targeting peer social skills, as early empathy training appears vital for social and emotional growth when children navigate social conflicts and relationships [2]. Forming close friendships and adapting to social norms are key developmental tasks during this stage [56], with growth partly driven by social learning opportunities at home, including modeling and reinforcement [57]. Our results demonstrate that relatively brief interventions during this time-period can produce measurable changes in creativity and empathy.

Measurement of empathy and creativity can be nuanced and complex, but there is some evidence societal levels are dropping [4,5,58,59]. This study provides more evidence that a simple bedtime reading routine can boost these key life skills. Unfortunately, reading activity levels and reading enjoyment levels have also dramatically and consistently dropped since 2010 [60,61]. Parents are aware that reading to their children has benefits, but further research should explore the broader implications of these findings for parenting and education, particularly in an increasingly digital age. This is particularly important for child development as the rise of artificial intelligence technologies makes the skills of creativity and empathy even more valuable assets for humanity.

### Study limitations

The finding that both reading groups showed similar improvements in empathy and creativity, regardless of the reflection intervention, suggests several alternative explanations for our results. Study participants watched a visual model showing a mother reading to a child on her lap, and research indicates that fostering intimacy through positive touch and close physical presence during reading sessions can strengthen emotional connections and enhance empathy, and this quality time seemed beneficial for both groups. Rather than reflection-specific benefits, the observed changes may reflect the inherent value of structured, daily reading experiences that provide repeated exposure to character emotions and social situations embedded within narrative contexts. The lack of differential effects between groups could indicate that children naturally engage in perspective-taking while listening to stories, making explicit prompting redundant during this developmental period.

Our study has several important limitations that must be acknowledged. Most critically, we lacked a no-reading control group, which limits our ability to make causal inferences about the effects of reading itself. Without a control group that did not receive books or reading instructions, we cannot definitively separate the effects attributable to reading from other factors such as natural developmental changes over the two-week period, practice effects from taking the empathy and creativity measures twice, or increased parent-child interaction time regardless of reading content.

The decision not to include a no-reading control was driven by several factors: 1) resource constraints limited us to approximately 40 families, and splitting these across three groups would have substantially reduced statistical power for detecting differences between our two reading groups, 2) recruitment and retention would have been more challenging for a no-reading group, particularly since families expressed interest specifically because they wanted their children to receive books, 3) our primary research question focused on the incremental benefit of adding reflection rather than comparing reading versus no reading, and 4) ethical considerations made it difficult to justify asking families to abstain from reading for two weeks given the well-documented benefits of parent-child reading. This design limitation means our findings may simply demonstrate that adding reflective questions to reading may provide incremental benefits beyond reading alone.

Additionally, our sample was predominantly from high-income families (92.11% earning > $50,000 annually), which substantially limits the generalizability of our findings to more diverse socioeconomic populations where access to books, reading habits, and family literacy practices may differ significantly. The relatively small child sample size (N = 38) further constrains our conclusions, as post-study power analysis [62] revealed sufficient (80%) power to detect only medium to large effects (partial R^2^ ≥ 18%) [63], but may have been underpowered to detect smaller but potentially meaningful changes. The absence of significant treatment × occasion interactions for empathy measures could reflect either a) truly similar effects of both reading approaches on empathy development, or b) smaller effect sizes that our study lacked sufficient power to detect. Given that both groups showed improvements in empathy from baseline to follow-up, it is possible that structured bedtime reading benefits empathy development regardless of whether reflection questions are included, but that our study was underpowered to detect subtle differences in the magnitude of these improvements.

The brief two-week intervention period may not have provided sufficient time for more robust empathy and creativity changes to emerge, particularly for emotional empathy, which research suggests requires longer periods to modify. Furthermore, our simplified empathy scale (Yes/No/Sometimes) and the constrained range of possible scores (0–6 for subscales) may have created ceiling and floor effects that obscured more nuanced changes in empathetic responding. Given these methodological constraints, these findings should be interpreted as preliminary evidence suggesting that consistent reading practices may support empathy and creativity development in young children, rather than definitive proof of causal mechanisms.

### Future directions

Future research should address the methodological limitations identified in this study through more rigorous experimental designs. Most importantly, studies should include a true control group that receives no reading intervention, as well as additional comparison conditions such as a group receiving equivalent attention without books (such as conversation time) to isolate the specific effects of literary exposure. Additionally, longer intervention periods (6–12 weeks) would allow researchers to examine whether empathy and creativity benefits persist beyond initial novelty effects and whether reflection-based approaches show delayed benefits that emerge with sustained practice. Future investigations should also examine if observed benefits persist when reading books without a social conflict component or if reading physical books together has any advantage over reading books on an electronic device.

Measurement improvements are also needed, including the use of validated empathy scales with broader response ranges, behavioral measures of empathetic responding (not just self-report), and multiple creativity assessments to avoid fluency contamination effects. Future studies should systematically examine individual difference factors such as socioeconomic status, cultural background, neurodevelopmental differences, and developmental readiness that may moderate intervention effectiveness. Investigation of different types of books (varying in social conflict complexity, character diversity, and narrative structure) would help identify optimal literary content for empathy development while retaining a fun, connective experience for families. Finally, longitudinal follow-up assessments would determine whether observed benefits maintain over time and translate into real-world prosocial behaviors and creative problem-solving abilities.

Examining the roles of adult interactive scaffolding, presence of physical touch, or the amount of intentional time together will help develop best practices recommendations for parents and educators, teaching them how to boost skills of empathy and creativity in young children. At a time when empathy and creativity are declining in society, this research suggests that something as simple and accessible as daily bedtime reading may offer families a powerful tool to nurture these essential human capacities in the next generation.

## Supporting information

S1 Fig Histograms of residuals for all empathy and creativity measures for the five primary ANOVA models. All distributions except emotional empathy and originality appeared symmetrical and visually demonstrated that the assumption of normality was met. Shapiro-Wilk and Levene’s tests were performed to confirm the assumptions of normality and homoscedasticity were met, and no power transformations on the data were deemed necessary to use for any measures.
(TIFF)

S1 Table Mixed ANOVA results, with sex as a moderator. (DOCX)

S2 Table Mixed ANOVA results, with age as a moderator. (DOCX)

S3 Table Mixed ANOVA results, with prior reading as a moderator. (DOCX)

S4 Table Cumulative link mixed effects model results, with sex, age, and prior reading as moderators. (DOCX)

S1 Video Examples_Winter2026. (MOV)
