# Supplementary figures and images for: Keep the bedtime story: A daily reading ritual improves empathy and creativity in children
Source: PLoS One. 2026 Jan 9;21(1):e0340068. doi: 10.1371/journal.pone.0340068 (PMC12788668; doi:10.1371/journal.pone.0340068)

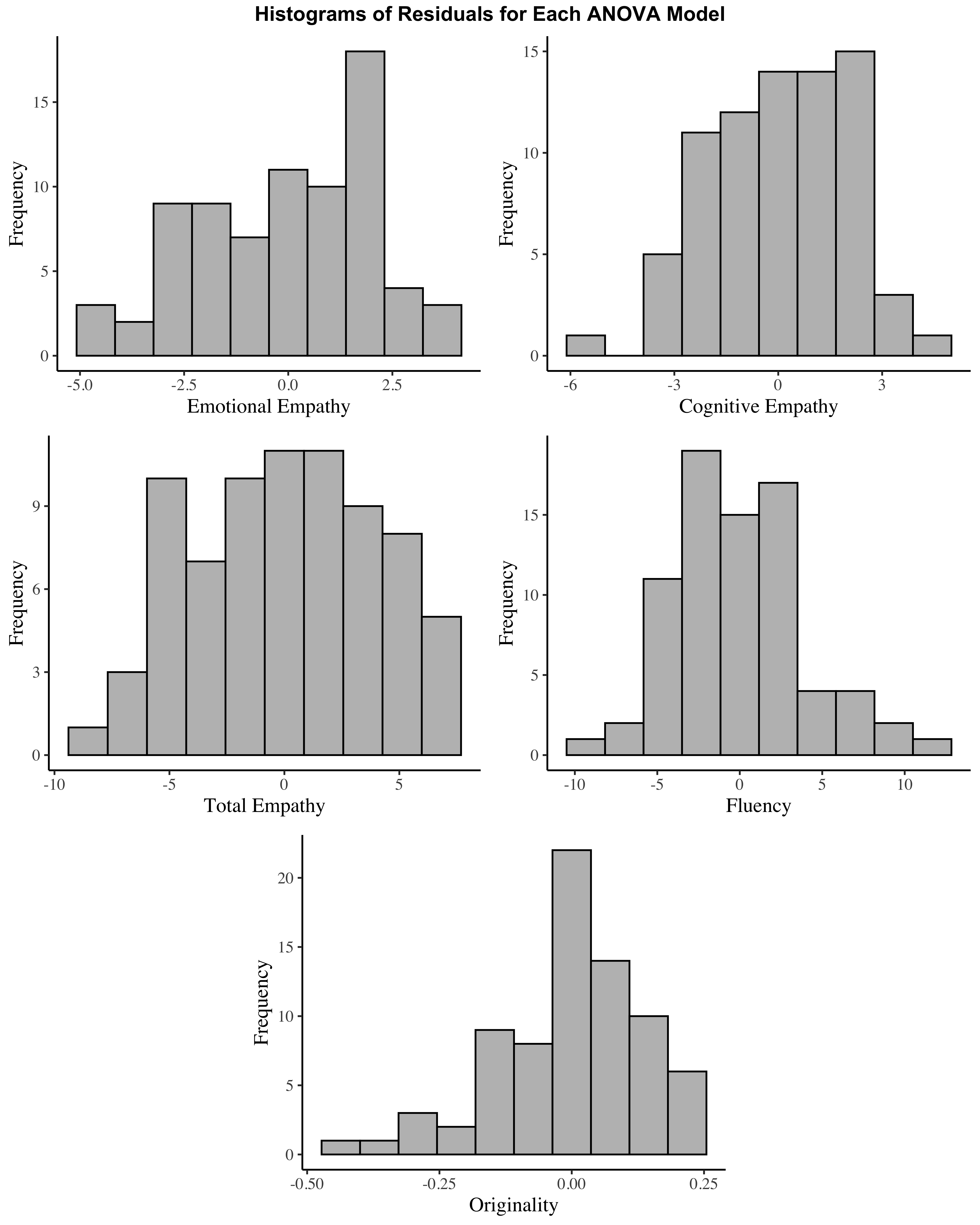

Supplement: S1 Fig — All distributions except emotional empathy and originality appeared symmetrical and visually demonstrated that the assumption of normality was met. Shapiro-Wilk and Levene’s tests were performed to confirm the assumptions of normality and homoscedasticity were met, and no power transformations on the data were deemed necessary to use for any measures. (TIFF) [file pone.0340068.s001.tiff]
