# Supplementary material for: Keep the bedtime story: A daily reading ritual improves empathy and creativity in children
Source: PLoS One. 2026 Jan 9;21(1):e0340068. doi: 10.1371/journal.pone.0340068 (PMC12788668; doi:10.1371/journal.pone.0340068)
Supplement: S1 Table — (DOCX) [file pone.0340068.s002.docx]

**Supplemental Table 1
Mixed ANOVA Results, with Sex as a Moderator**

| **Measures** | **Effect** | **F-Value** | **p-value** | **Corrected**  **p-value** | $\boldsymbol{R}_{\boldsymbol{p}}^{\boldsymbol{2}}$ |
| --- | --- | --- | --- | --- | --- |
| **Empathy** |  |  |  |  |  |
| Emotional | Treatment (Read Through vs. Pausing) | 2.18 | .149 | .596 | 0.06 |
|  | Occasion (Initial vs. Follow-Up) | 1.41 | .243 | .972 | 0.04 |
|  | Sex (Male vs. Female) | 0.58 | .450 | > .999 | 0.02 |
|  | Treatment x Occasion Interaction | 0.13 | .717 | > .999 | < 0.01 |
|  | Treatment x Sex Interaction | 0.38 | .544 | > .999 | 0.01 |
|  | Occasion x Sex Interaction | 0.13 | .717 | > .999 | < 0.01 |
|  | Treatment x Occasion x Sex Interaction | 0.21 | .651 | > .999 | 0.01 |
| Cognitive | Treatment (Read Through vs. Pausing) | 1.32 | .259 | > .999 | 0.04 |
|  | Occasion (Initial vs. Follow-Up) | 8.69 | .006** | .024* | 0.20 |
|  | Sex (Male vs. Female) | 1.52 | .227 | .908 | 0.04 |
|  | Treatment x Occasion Interaction | 2.43 | .128 | .512 | 0.07 |
|  | Treatment x Sex Interaction | 5.09 | .031* | .124 | 0.13 |
|  | Occasion x Sex Interaction | 1.34 | .255 | > .999 | 0.04 |
|  | Treatment x Sex x Occasion Interaction | 0.02 | .897 | > .999 | < 0.01 |
| Total | Treatment (Read Through vs. Pausing) | 2.47 | .126 | .504 | 0.07 |
|  | Occasion (Initial vs. Follow-Up) | 8.10 | .008** | .032* | 0.20 |
|  | Sex (Male vs. Female) | 0.59 | .446 | > .999 | 0.02 |
|  | Treatment x Occasion Interaction | 4.76 | .036* | .144 | 0.13 |
|  | Treatment x Sex Interaction | 4.04 | .053 | .212 | 0.11 |
|  | Occasion x Sex Interaction | 0.44 | .511 | > .999 | 0.01 |
|  | Treatment x Sex x Occasion Interaction | 1.30 | .263 | > .999 | 0.04 |
| **Creativity** |  |  |  |  |  |
| Fluency | Treatment (Read Through vs. Pausing) | 0.01 | .914 | > .999 | < 0.01 |
|  | Occasion (Initial vs. Follow-Up) | 22.00 | < .001*** | < .001*** | 0.39 |
|  | Sex (Male vs. Female) | 1.53 | .225 | .450 | 0.04 |
|  | Treatment x Occasion Interaction | 5.92 | .020* | .040* | 0.15 |
|  | Treatment x Sex Interaction | 0.02 | .903 | > .999 | < 0.01 |
|  | Occasion x Sex Interaction | 3.61 | .066 | .132 | 0.10 |
|  | Treatment x Sex x Occasion Interaction | 0.30 | .585 | > .999 | 0.01 |
| Originality | Treatment (Read Through vs. Pausing) | 0.42 | .520 | > .999 | 0.01 |
|  | Occasion (Initial vs. Follow-Up) | 79.65 | < .001*** | < .001*** | 0.70 |
|  | Sex (Male vs. Female) | 3.14 | .085 | .170 | 0.08 |
|  | Treatment x Occasion Interaction | 0.75 | .392 | .784 | 0.02 |
|  | Treatment x Sex Interaction | 0.09 | .770 | > .999 | <0.01 |
|  | Occasion x Sex Interaction | 0.01 | .921 | > .999 | <0.01 |
|  | Treatment x Sex x Occasion Interaction | 1.15 | .291 | .582 | 0.03 |

Note: The F-values for Total Empathy have 1 and 33 degrees of freedom, and all other F-values have 1 and 34 degrees of freedom. Rows that are statistically significant, post-correction, are shaded green. Corrected p-values are based on a Bonferroni adjustment, assuming a familywise error rate of four comparisons for models that focused on the four empathy measures, and two comparisons for the two creativity measures. The number of stars after each p-value represent the level of statistical significance; *: $.01\leq p<.05$; **: $.001\leq p<.01$; ***: $p<.001$.
