# Supplementary material for: Keep the bedtime story: A daily reading ritual improves empathy and creativity in children
Source: PLoS One. 2026 Jan 9;21(1):e0340068. doi: 10.1371/journal.pone.0340068 (PMC12788668; doi:10.1371/journal.pone.0340068)
Supplement: S2 Table — (DOCX) [file pone.0340068.s003.docx]

**Supplemental Table 2
Mixed ANOVA Results, with Age as a Moderator**

| **Measures** | **Effect** | **F-Value** | **p-value** | **Corrected**  **p-value** | $\boldsymbol{R}_{\boldsymbol{p}}^{\boldsymbol{2}}$ |
| --- | --- | --- | --- | --- | --- |
| **Empathy** |  |  |  |  |  |
| Emotional | Treatment (Read Through vs. Pausing) | 1.69 | .203 | .812 | 0.05 |
|  | Occasion (Initial vs. Follow-Up) | 0.50 | .487 | > .999 | 0.02 |
|  | Age | 1.26 | .296 | > .999 | 0.07 |
|  | Treatment x Occasion Interaction | <0.01 | .976 | > .999 | < 0.01 |
|  | Treatment x Age Interaction | 0.11 | .896 | > .999 | 0.01 |
|  | Occasion x Age Interaction | 0.24 | .791 | > .999 | 0.02 |
|  | Treatment x Occasion x Age Interaction | 0.50 | .614 | > .999 | 0.03 |
| Cognitive | Treatment (Read Through vs. Pausing) | 1.73 | .197 | .788 | 0.05 |
|  | Occasion (Initial vs. Follow-Up) | 5.63 | .024* | .096 | 0.15 |
|  | Age | 0.41 | .664 | > .999 | 0.02 |
|  | Treatment x Occasion Interaction | 1.78 | .191 | .764 | 0.05 |
|  | Treatment x Age Interaction | 0.69 | .509 | > .999 | 0.04 |
|  | Occasion x Age Interaction | 0.68 | .515 | > .999 | 0.04 |
|  | Treatment x Occasion x Age Interaction | 0.16 | .856 | > .999 | 0.01 |
| Total | Treatment (Read Through vs. Pausing) | 2.46 | .127 | .508 | 0.07 |
|  | Occasion (Initial vs. Follow-Up) | 4.30 | .046* | .184 | 0.12 |
|  | Age | 0.71 | .499 | > .999 | 0.04 |
|  | Treatment x Occasion Interaction | 2.02 | .165 | .660 | 0.06 |
|  | Treatment x Age Interaction | 0.32 | .726 | > .999 | 0.02 |
|  | Occasion x Age Interaction | 0.56 | .576 | > .999 | 0.04 |
|  | Treatment x Occasion x Age Interaction | 0.76 | .476 | > .999 | 0.05 |
| **Creativity** |  |  |  |  |  |
| Fluency | Treatment (Read Through vs. Pausing) | 0.02 | .905 | > .999 | < 0.01 |
|  | Occasion (Initial vs. Follow-Up) | 14.74 | < .001 | .001 | 0.32 |
|  | Age | 0.50 | .613 | > .999 | 0.03 |
|  | Treatment x Occasion Interaction | 4.36 | .045* | .090 | 0.12 |
|  | Treatment x Age Interaction | 0.65 | .528 | > .999 | 0.04 |
|  | Occasion x Age Interaction | 0.78 | .465 | .930 | 0.05 |
|  | Treatment x Occasion x Age Interaction | 0.01 | .995 | > .999 | < 0.01 |
| Originality | Treatment (Read Through vs. Pausing) | 0.03 | .856 | > .999 | < 0.01 |
|  | Occasion (Initial vs. Follow-Up) | 63.56 | < .001*** | < .001*** | 0.66 |
|  | Age | 5.45 | .009** | .018* | 0.25 |
|  | Treatment x Occasion Interaction | 0.65 | .425 | .850 | 0.02 |
|  | Treatment x Age Interaction | 1.14 | .334 | .668 | 0.07 |
|  | Occasion x Age Interaction | 0.94 | .403 | .806 | 0.06 |
|  | Treatment x Occasion x Age Interaction | 0.22 | .805 | > .999 | 0.01 |

Note: The F-values for Total Empathy have 1 and 31 degrees of freedom, and all other F-values have 1 and 32 degrees of freedom. Rows that are statistically significant, post-correction, are shaded green. Corrected p-values are based on a Bonferroni adjustment, assuming a familywise error rate of four comparisons for models that focused on the four empathy measures, and two comparisons for the two creativity measures. The number of stars after each p-value represent the level of statistical significance; *: $.01\leq p<.05$; **: $.001\leq p<.01$; ***: $p<.001$.
