# Supplementary material for: Keep the bedtime story: A daily reading ritual improves empathy and creativity in children
Source: PLoS One. 2026 Jan 9;21(1):e0340068. doi: 10.1371/journal.pone.0340068 (PMC12788668; doi:10.1371/journal.pone.0340068)
Supplement: S3 Table — (DOCX) [file pone.0340068.s004.docx]

**Supplemental Table 3
Mixed ANOVA Results, with Prior Reading as a Moderator**

| **Measures** | **Effect** | **F-Value** | **p-value** | **Corrected**  **p-value** | $\boldsymbol{R}_{\boldsymbol{p}}^{\boldsymbol{2}}$ |
| --- | --- | --- | --- | --- | --- |
| **Empathy** |  |  |  |  |  |
| Emotional | Treatment (Read Through vs. Pausing) | 1.88 | .179 | .716 | 0.05 |
|  | Occasion (Initial vs. Follow-Up) | 1.48 | .232 | .928 | 0.04 |
|  | Prior Reading (Beginner vs. Independent) | 0.98 | .329 | > .999 | 0.03 |
|  | Treatment x Occasion Interaction | 0.07 | .799 | > .999 | < 0.01 |
|  | Treatment x Prior Reading Interaction | 1.02 | .320 | > .999 | 0.03 |
|  | Occasion x Prior Reading Interaction | 1.29 | .265 | > .999 | 0.04 |
|  | Treatment x Occasion x Prior Reading | 0.24 | .626 | > .999 | 0.01 |
| Cognitive | Treatment (Read Through vs. Pausing) | 0.78 | .383 | > .999 | 0.02 |
|  | Occasion (Initial vs. Follow-Up) | 8.18 | .007** | .028* | 0.19 |
|  | Prior Reading (Beginner vs. Independent) | 1.86 | .181 | .724 | 0.05 |
|  | Treatment x Occasion Interaction | 2.38 | .132 | .528 | 0.06 |
|  | Treatment x Prior Reading Interaction | 0.06 | .802 | > .999 | < 0.01 |
|  | Occasion x Prior Reading Interaction | 0.12 | .737 | > .999 | < 0.01 |
|  | Treatment x Occasion x Prior Reading | 0.01 | .934 | > .999 | < 0.01 |
| Total | Treatment (Read Through vs. Pausing) | 1.76 | .194 | .776 | 0.05 |
|  | Occasion (Initial vs. Follow-Up) | 8.12 | .007** | .028* | 0.20 |
|  | Prior Reading (Beginner vs. Independent) | 3.26 | .080 | .320 | 0.09 |
|  | Treatment x Occasion Interaction | 4.42 | .043* | .172 | 0.12 |
|  | Treatment x Prior Reading Interaction | 0.05 | .822 | > .999 | < 0.01 |
|  | Occasion x Prior Reading Interaction | 0.30 | .589 | > .999 | 0.01 |
|  | Treatment x Occasion x Prior Reading | 0.14 | .716 | > .999 | < 0.01 |
| **Creativity** |  |  |  |  |  |
| Fluency | Treatment (Read Through vs. Pausing) | 0.03 | .872 | > .999 | < 0.01 |
|  | Occasion (Initial vs. Follow-Up) | 19.34 | < .001*** | < .001*** | 0.36 |
|  | Prior Reading (Beginner vs. Independent) | 2.94 | .096 | .192 | 0.08 |
|  | Treatment x Occasion Interaction | 5.68 | .023* | .046* | 0.14 |
|  | Treatment x Prior Reading Interaction | 0.25 | .623 | > .999 | 0.01 |
|  | Occasion x Prior Reading Interaction | 0.01 | .928 | > .999 | < 0.01 |
|  | Treatment x Occasion x Prior Reading | 0.01 | .912 | > .999 | < 0.01 |
| Originality | Treatment (Read Through vs. Pausing) | 0.06 | .807 | > .999 | 0.70 |
|  | Occasion (Initial vs. Follow-Up) | 77.73 | < .001*** | < .001*** | 0.19 |
|  | Prior Reading (Beginner vs. Independent) | 8.13 | .007** | .014* | 0.02 |
|  | Treatment x Occasion Interaction | 0.74 | .397 | .794 | 0.02 |
|  | Treatment x Prior Reading Interaction | 0.82 | .372 | .744 | 0.02 |
|  | Occasion x Prior Reading Interaction | 0.53 | .473 | .946 | 0.04 |
|  | Treatment x Occasion x Prior Reading | 1.24 | .274 | .548 | < 0.01 |

Note: The F-values for Total Empathy have 1 and 33 degrees of freedom, and all other F-values have 1 and 34 degrees of freedom. Rows that are statistically significant, post-correction, are shaded green. Corrected p-values are based on a Bonferroni adjustment, assuming a familywise error rate of four comparisons for models that focused on the four empathy measures, and two comparisons for the two creativity measures. The number of stars after each p-value represent the level of statistical significance; *: $.01\leq p<.05$; **: $.001\leq p<.01$; ***: $p<.001$.
