# Supplementary material for: Keep the bedtime story: A daily reading ritual improves empathy and creativity in children
Source: PLoS One. 2026 Jan 9;21(1):e0340068. doi: 10.1371/journal.pone.0340068 (PMC12788668; doi:10.1371/journal.pone.0340068)
Supplement: S4 Table — (DOCX) [file pone.0340068.s005.docx]

**Supplemental Table 4
Cumulative Link Mixed Effects Model Results, with Sex, Age, and Prior Reading as Moderators**

| **Model** | **Effect** | **Slope** | **Z-Value** | **p-value** | **Corrected**  **p-value** | $\boldsymbol{R}_{\boldsymbol{p}}^{\boldsymbol{2}}$  **(McFadden)** | $\boldsymbol{R}_{\boldsymbol{p}}^{\boldsymbol{2}}$  **(Nagelkerke)** |
| --- | --- | --- | --- | --- | --- | --- | --- |
| **Sex as Moderator** |  |  |  |  |  |  |  |
| Fantasy | Treatment (Read Through vs. Pausing) | 0.44 | 1.78 | .075 | .300 | 0.01 | 0.04 |
|  | Occasion (Initial vs. Follow-Up) | -0.42 | -1.63 | .103 | .412 | 0.02 | 0.05 |
|  | Sex (Male vs. Female) | -0.23 | -0.98 | .329 | > .999 | < 0.01 | 0.01 |
|  | Treatment x Occasion Interaction | 0.71 | 2.63 | .008** | .034* | 0.04 | 0.12 |
|  | Treatment x Sex Interaction | -0.29 | -1.25 | .213 | .850 | 0.01 | 0.02 |
|  | Occasion x Sex Interaction | 0.23 | 1.00 | .318 | > .999 | 0.01 | 0.02 |
|  | Treatment x Occasion x Sex Interaction | -0.38 | -1.48 | .138 | .554 | 0.01 | 0.04 |
| **Age as Moderator** |  |  |  |  |  |  |  |
| Fantasy | Treatment (Read Through vs. Pausing) | -0.55 | -0.26 | .797 | > .999 | 0.01 | 0.03 |
|  | Occasion (Initial vs. Follow-Up) | -0.63 | -0.29 | .769 | > .999 | 0.02 | 0.05 |
|  | Age | -0.12 | 0.39 | .699 | > .999 | < 0.01 | < 0.01 |
|  | Treatment x Occasion Interaction | 2.06 | 0.94 | .348 | > .999 | 0.04 | 0.11 |
|  | Treatment x Age Interaction | 0.14 | 0.44 | .663 | > .999 | < 0.01 | < 0.01 |
|  | Occasion x Age Interaction | 0.03 | 0.11 | .915 | > .999 | < 0.01 | < 0.01 |
|  | Treatment x Occasion x Age Interaction | -0.21 | -0.65 | .514 | > .999 | < 0.01 | 0.01 |
| **Prior Reading as Moderator** |  |  |  |  |  |  |  |
| Fantasy | Treatment (Read Through vs. Pausing) | 0.41 | 1.67 | .094 | .376 | 0.01 | 0.03 |
|  | Occasion (Initial vs. Follow-Up) | -0.44 | -1.76 | .079 | .314 | 0.02 | 0.05 |
|  | Prior Reading (Beginner vs. Independent) | -0.24 | -1.00 | .317 | > .999 | < 0.01 | 0.01 |
|  | Treatment x Occasion Interaction | 0.72 | 2.60 | .009** | .038* | 0.04 | 0.12 |
|  | Treatment x Prior Reading Interaction | -0.36 | -1.46 | .143 | .572 | 0.01 | 0.03 |
|  | Occasion x Prior Reading Interaction | 0.15 | 0.63 | .531 | > .999 | < 0.01 | 0.01 |
|  | Treatment x Occasion x Prior Reading | -0.03 | -0.12 | .905 | > .999 | < 0.01 | < 0.01 |

Note: Rows that are statistically significant, post-correction, are shaded green. Corrected p-values are based on a Bonferroni adjustment, assuming a familywise error rate of four comparisons for models that focused on the four empathy measures (see Table 3 for the other three empathy measure results). The reported $\boldsymbol{R}_{\boldsymbol{p}}^{\boldsymbol{2}}$ values are McFadden and Nagelkerke pseudo R-square measures, respectively, computed using Type II model comparisons. The number of stars after each p-value represent the level of statistical significance; *: $.01\leq p<.05$; **: $.001\leq p<.01$; ***: $p<.001$.
